# Individual and group level personality change across the lifespan in dogs

**DOI:** 10.1038/s41598-020-74310-7

**Published:** 2020-10-14

**Authors:** Borbála Turcsán, Lisa Wallis, Judit Berczik, Friederike Range, Enikő Kubinyi, Zsófia Virányi

**Affiliations:** 1 Clever Dog Lab, Comparative Cognition, Messerli Research Institute, University of Veterinary Medicine, Vienna, Medical University of Vienna, and University of Vienna, Vienna, Austria; 2 Department of Ethology, ELTE Eötvös Loránd University, Budapest, Hungary; 3 Department of Livestock and One Health, Institute of Infection, Veterinary and Ecological Sciences, University of Liverpool, Liverpool, UK

**Keywords:** Ageing, Animal behaviour

## Abstract

In humans, age-related changes in personality occur in a non-random fashion with respect to their direction, timing, and magnitude. In dogs, there are still gaps in our knowledge about the detailed dynamics of age-related personality changes. We analysed the personality of 217 Border collies aged from 0.5 to 15 years both cross-sectionally and longitudinally using a test battery, to specify age periods when changes most prominently occur, assess the magnitude of changes, and analyse individual differences in personality change. We found that similar to humans, changes in personality occur unevenly during the dogs’ life course, however, their dynamics seems to be specific for each trait. Activity-independence decreased mostly from puppyhood (0.5–1 years) to adolescence (> 1–2 years), then continued to decrease in a slowing rate. Novelty seeking did not change markedly until middle age (> 3–6 years), then showed a steady linear decrease. Problem orientation increased strongly until middle age then showed no marked changes in later age periods. We also revealed individual differences in personality change over time, and showed that a few individuals with potential age-related impairments significantly affected the general age trajectory of some traits. These results raise caution against the over-generalisation of global age trends in dogs.

## Introduction

A core component of the concept of personality is that individuals differ in suites of correlated behaviours consistently over time^1^. Temporal consistency, however, does not equate with temporal stability, meaning that personality can and does change with age. As such, in humans, even if personality differences across individuals are maintained over the majority of the lifespan (even after 50 years^2^), the trait scores of the individuals change with age in a near-universal pattern, commonly referred to as personality maturation: older people become more conscientious, agreeable, emotionally stable, and dominant^2–4^. The dynamics of personality change also follow a non-random pattern (referred to as the cumulative continuity principle): while personality changes throughout the whole lifespan, the majority of changes occur in adolescence and young adulthood (until ~ 30–40 years of age), whereas changes become more gradual and modest at later ages^4, 5^. But why does personality change at all? Some researchers hypothesize that personality development is genetically pre-programmed, similar to cognitive maturation in children or to the menopause^6^. This hypothesis could explain the universality of personality changes found across many different cultures^7^ and it is, to some extent, supported by twin studies^8^ and human-parallel personality changes observed in chimpanzees^9^. Others advocate that personality changes in response to life events and transitions, to facilitate successful integration into new roles (like becoming parents, productive workers, etc.)^10–12^. This hypothesis is supported by accumulating evidence for strong individual variability in the direction and rate of personality change over time^5^. For example, younger people with a more ‘mature’ personality profile are less likely to change with age^13^, as they are already better equipped to deal with social-developmental challenges across the life course^3^. Thus, there are still several open questions about human personality development, including how biological (genetic, hormonal) and external factors (environment, life experiences) contribute to the direction, timing, and magnitude of personality change over time^3, 5, 12^.

Animal models could shed light on the mechanisms that drive human personality development^9^, and among them, domestic dogs are increasingly recognized as a natural model for human ageing, including both molecular (genetic) markers^14^, and phenotypic manifestation^15, 16^. Therefore, we might expect that similar rules govern the ageing processes of dogs and humans, including the ageing dynamics of personality. While dogs may not be pressed by human society to adapt into new roles at different life stages, owners actively shape their dogs’ behaviour via training, especially at a young age, implying that they would expect (and tolerate) different behaviours from puppies or adolescent dogs than from adults or aged dogs. The daily routines of dogs also change with age, especially with regards to different shared activities with their owners (i.e., older dogs receive less training, off-leash activity and play^17, 18^). Older dogs are also more likely to experience several fundamental life events (e.g., change in family structure, moving to a new house, or traumas) than younger dogs (based on data provided in^17^). All these changes may well contribute to age-related changes in personality in dogs.

Even though dog personality is a highly popular topic in the literature, some details have so far remained largely unexplored. Here we identify four main gaps in our relevant knowledge.

1. *Lifespan* *trajectory* *of* *personality* *development* *in* *dogs* While numerous studies have reported age-related associations in various dog personality traits on cross-sectional samples^19–21^, studies have rarely investigated the dynamics of the age trajectory of personality traits, that is, when these changes occur. This is a problem because some studies have revealed that the relationship between age and behaviour is not always linear^22, 23^, so dog ageing studies which compared only young and aged dogs or analysed the effect of age only with correlations or with linear models, may have led to biased conclusions.
2. *Impaired* *dogs* A proportion of aged dogs, especially those considered senior/geriatric could suffer from age-related physical, sensory, or mental impairments which could change their behaviour compared to that of successfully ageing dogs^24, 25^. Studies assessing behaviours other than cognition rarely make efforts to identify potentially impaired dogs in their sample, even though individuals with severe behavioural deterioration may affect the general age trajectory of a given personality trait.
3. *Mean-level* *change:* Previous dog personality studies have rarely (if at all) quantified the magnitude of trait change (mean-level change) over time (but see^21^). Thus, there is almost no knowledge of how much individuals actually change throughout their life course, and in which life stages the most prominent changes in personality occur.
4. *Individual* *differences* *in* *personality* *change* A significant age-personality association does not necessarily mean that all individuals change, or that their traits change in the same direction. Similarly, when there is no significant age-personality association there could still be subsets of individuals that show an increase or decrease in trait scores. While individual differences in personality change are keystones in personality development theories^5^, very few studies attempted to account for individual differences in behavioural changes in dogs (but see^26, 27^), and longitudinal analyses over several years are completely lacking.

Filling these gaps is crucial for understanding the personality development of dogs through their lifespan. This can not only enable drawing better parallels with humans but has also important practical relevance. Behavioural changes, even those indicative of age-related diseases, are commonly dismissed by owners as part of the normal ageing process^28, 29^. Understanding what is considered a normal age-related change in personality, regarding its timing, direction and magnitude, can help the early identification of impairments^30, 31^.

In the current study, we carried out a comprehensive analysis of age-related changes in dog personality using test data from > 200 Border collies across a wide age range (from 0.5 to 15 years). In particular, we aimed to:

1. investigate the *general* *age* *trajectory* of five personality traits over the majority of the dogs’ life course, testing for both linear and quadratic relationships;
2. identify *potentially* *impaired* *individuals* among the senior and geriatric dogs (> 8 years of age) based on their behaviour, and investigate if/how much they affect the general age trajectory of the entire sample;
3. quantify the magnitude of personality trait differences across age groups (*mean-level* *change*) and identify at what age changes are most prominent;
4. analyse personality *change* *at* *the* *individual* *level* *in* *a* *longitudinal* *study* and investigate whether individuals with different initial personality profiles show similar or different changes after a ~ 4-year-long period.

For the first three aims, we used a cross-sectional approach. While these analyses were mainly explorative and descriptive in nature, we expected that, in accordance with the cumulative continuity principle in humans^4^, personality would most prominently change until the end of middle age (~ 6 years of age) after which the rate of change would slow down in senior and geriatric ages. Regarding our fourth aim, we expected that, similar to humans, dogs with a more ‘mature’ initial personality profile (based on the results of the cross-sectional analyses and their age at first testing) would change less than others.

## Methods

### Ethical statement

The conducted research was based on non-invasive procedures to assess dogs’ behaviour, and such non-invasive observational studies can be conducted without any special permission in Austria (Tierversuchsgesetz 2012–TVG 2012). The experimental procedure was discussed and approved by the institutional ethics and animal welfare committee at the University of Veterinary Medicine Vienna (Approval numbers: 09/04/97/2012, 04/05/97/2012, 09/10/97/2012, 09/06/2015) in accordance with Good Scientific Practice guidelines and national legislation. The owners participated in the test voluntarily. They were informed about the purpose and procedure of the test beforehand, and they all signed an informed consent form permitting their dogs to participate in the study and allowing us to use the recorded data in publications.

### Subjects

Cross-sectional sample: 220 Border collies participated in the study, recruited among volunteers of the Clever Dog Database in Vienna, Austria. Three dogs were excluded from this sample, two because of the malfunctioning video equipment, and one because she was stressed and not willing to approach the experimenter, so the test could not be carried out. In the final sample (N = 217), the dogs’ age ranged from 0.5 to 15 years (mean ± SD = 4.0 ± 3.5), and 56.7% (N = 123) were females.

Longitudinal sample: 4 years after the cross-sectional sampling, we contacted the owners of all dogs who were still alive when the re-testing was done and had participated in no other study using methods similar to our tests. Altogether 37 dogs were available for re-testing. 56.8% of the dogs (N = 21) were females, the dogs’ age at their first test session ranged from 0.5 to 7.1 years (mean ± SD = 2.8 ± 1.9 years), and from 3.5 to 11.3 years (mean ± SD = 6.5 ± 2.0 years) at their second test session. The time interval between the two test sessions ranged from 2.5 to 4.7 years (mean: 3.8 years). The detailed distribution of the dogs in both samples according to different age periods is presented in the Supplementary Table 1.

**Table 1:** Relationship between the five personality traits and the age of the dogs in the full sample, and after excluding four outlier aged dogs.

Personality trait	N	Regression	Full sample			Outliers excluded		
			R^2^	F	*p* value	R^2^	F	*p*
Sociability-obedience	215	Linear	0.004	0.803	0.371	0.000	0.015	0.903
		Quadratic	0.015	1.594	0.205	0.000	0.034	0.967
Activity-independence	210	Linear	0.006	1.308	0.254	0.071	15.601	< 0.001
		Quadratic	0.143	17.309	< 0.001	0.107	12.142	< 0.001
Novelty seeking	215	Linear	0.310	95.604	< 0.001	0.330	103.234	< 0.001
		Quadratic	0.318	49.458	< 0.001	0.332	51.824	< 0.001
Problem orientation	217	Linear	0.105	25.154	< 0.001	0.161	40.626	< 0.001
		Quadratic	0.185	24.356	< 0.001	0.198	25.975	< 0.001
Frustration tolerance	188	Linear	0.027	5.167	0.024	0.026	4.952	0.027
		Quadratic	0.028	2.621	0.075	0.027	2.522	0.083

The results of both the linear and quadratic regressions are shown.

### Study design

The first test sessions used in the cross-sectional analyses were conducted by one of three female experimenters (all unfamiliar to the dogs) and were carried out indoors (room sizes: 5 × 6 m or 7.2 × 8 m). In our previous study^32^, we found no significant effect of the experimenter or test location on the dogs’ behaviour, so location and experimenter were not included in the current analyses.

The second test sessions used in the longitudinal analyses were conducted by one female experimenter (JB), who had not taken part in the first test sessions. These tests were carried out in a different test room of a similar size, to ensure that the room was also unfamiliar to the dogs. The experimental protocol was the same as in the first test session, but we used some experimental equipment (i.e., T-shirt, boxes, bags, umbrella, leash, and plates) with a different colour and material to minimize familiarity.

### Procedure

The test battery (‘VIDOPET’ Vienna Dog Personality Test^32^) consisted of 15 subtests with a short break (5–10 min) after the 7th subtest, and took ~ 1 h to complete. In both test sessions, the subtests were carried out in a predetermined order. For ethical and safety reasons, to minimise dogs fear responses during the test battery, we did not allow any situation to proceed in the case of a strong fear (or aggressive) reaction. All potentially negative or stressful situations were resolved immediately after they happened, and the testing was resumed only when the owner and dog were in a relaxed state. Here we provide a short description of the tests, the detailed protocol can be found in the Supplementary Methods (including descriptions of how potentially negative or stressful situations were resolved).

1. *Exploration* The dog could explore the room and different objects on the floor for 1 min, while the owner stood in the middle of the room ignoring the dog.
2. *Picture* *viewing* The owner walked slowly around the room while completely ignoring the dog for 1 min.
3. *Greeting* *the* *experimenter* The experimenter entered the room, approached the dog-owner pair in a friendly manner and petted the dog.
4. *Food* *choice* In phase 1 the dog could choose between an empty plate and a plate with a piece of food on it. In the second phase, the owner expressed a preference for the empty plate before the dog could, again, make a choice.
5. *Frustration* *test* The experimenter swung a large piece of sausage on a string in front of the dog’s nose (just out of the dog’s reach) for 1 min.
6. *Separation* The dog was left alone in the room for 1 min.
7. *Greeting* *after* *separation* The experimenter returned to the room, greeted the dog and played with it for 30 s, and then left the room. Afterwards the owner returned and repeated the same procedure.
8. *Problem* *I* (*cage*) In Trial 1, the dog had to completely pull out a string from a cage to get a piece of food. In Trial 2 (blocked trial) the string was fixed to the cage and the dog could interact with the cage for 5 min.
9. *T-shirt* *test* The owner dressed the dog in a T-shirt, then walked slowly around the room while completely ignoring the dog for 1 min.
10. *Obedience* *test* The owner gave four basic commands (sit, lay down, stay, come) to the dog while the experimenter was trying to distract the dog with rustling noises.
11. *Threatening* *approach* The experimenter approached the dog slowly, with a slightly bent upper body, staring steadily in the eyes of the dog, and without any verbal communication.
12. *Post-threat* *interaction* Following the threatening approach, E crouched down and called the dog in a friendly way.
13. *Problem* *II* (*bin*) The owner demonstrated to the dog how to remove the lid of a bin to get a piece of sausage from it, then the dog had 1 min. to remove the lid and get the food.
14. *Novel* *object* The dog encountered a self-moving toy which made a sound and had 1 min. to interact with it.
15. *Ball* *play* The owner threw a tennis ball three times and encouraged the dog to retrieve it. Then he/she stopped interacting with the dog and slowly walked around the room.

### Personality traits

All tests were video-taped and we coded 70 behaviour variables from the recordings using the Solomon Coder program (András Péter; www.solomoncoder.com). A detailed description of the method used to obtain the personality traits can be found in^32^. In short, a two-step data reduction method on the 70 variables (principal component analyses on the subtest level, followed by an exploratory factor analysis with Varimax rotation on the subtest components) was carried out on the data from the first test session of the dogs, and resulted in five traits, labelled as Sociability-obedience, Activity-independence, Novelty seeking, Problem orientation, and Frustration tolerance (Supplementary Table S2). In our previous study^32^ we demonstrated excellent inter- and intra-observer reliability and adequate internal consistency of all traits, and all traits showed significant test–retest reliability, and thus proved to be consistent over ~ 4 years.

### Statistical analyses

1. *Age trajectory across the lifespan* We investigated the age-personality relationship using linear and quadratic regression models. The dogs’ age was calculated as a year fraction between their date of birth and their date of testing.
2. *Impaired dogs* To identify potentially impaired individuals among the senior and geriatric dogs (> 8 years, N = 40), box plots were created for each of the five traits. The box plots identify outliers based on the interquartile range (IQR) and categorise outliers as values which are higher than the 75th percentile + 1.5 × IQR or lower than the 25th percentile—1.5 × IQR thresholds. This method is better suited for smaller samples as it is less sensitive to influences by extreme outliers than the more commonly used mean ± 3 SD threshold. We ran the linear and quadratic regression models again with the identified outliers excluded.
3. *Mean-level change* To quantify the mean-level personality change between the different age periods we assigned the dogs into seven age groups based on Wallis et al.^22^. This classification reflects the developmental periods in the Border collie: late puppyhood (0.5–1 years), adolescence (> 1–2 years), early adulthood (> 2–3 years), middle-age (> 3–6 years), late adulthood (> 6–8 years), senior age (> 8–10 years) and geriatric age (> 10 years). Since we expected that personality would change most prominently until the dogs reach middle age, we opted for younger age periods with shorter length in order to assess the direction and magnitude of change in sufficient detail. We compared the seven age groups in personality traits using one-way ANOVAs with Tukey posthoc tests if no violation of homoscedasticity was found, or Kruskal–Wallis tests if the variances differed between age groups. The trait scores had been transformed using square or square root transformation to ensure normality. We computed Hedges’ g to quantify mean-level change between the age groups and provide effect sizes of all group differences. Hedges’ g is a measure of standardized mean difference that also takes sample sizes into account, and is thus more accurate for smaller samples than Cohen’s d^33^. Hedges’ g = 1 indicates the two groups differ by 1 standard deviation.
4. *Individual differences in personality change* We compared the trait scores between the two test sessions using paired t-tests, and analysed how many dogs changed significantly from Test 1 to Test 2 for each trait. For this latter, we calculated the Reliable Change Index (RCI) for each individual, using the formula provided by Jacobson and Truax^34^. If the RCI value is greater than 1.96 or smaller than -1.96, then the change is significant; that is, presumably not the result of the unreliability of the measurement. The distribution of different types of dogs expected by chance would be 2.5% of dogs that showed an significant increase, 2.5% a decrease, and 95% that showed no significant change^2^. As our sample size is small, the numbers predicted as such are also small, so p-values of any goodness-of-fit statistics comparing the observed distribution to the distribution expected by chance would be inaccurate. Therefore, we provided only descriptive statistics of the distribution of the different types of dogs (those that significantly increased, decreased, or did not change significantly) for each trait. Finally, we also analysed the effect of the individual’s performance in the first test session on the magnitude of personality change from Test 1 to Test 2 using a similar method as described in Svartberg et al.^26^. We ranked the dogs according to their trait scores in the first test session and divided them into three groups: Low (first quartile), Intermediate (second and third quartile), and High (fourth quartile). We analysed whether the magnitude of change, calculated by subtracting the test score 1 from test score 2, was different between the three dog groups using one-way ANOVAs with Tukey post hoc tests.

SPSS 22 for Windows was used for all statistical analyses except for Hedges’ g and RCI that were calculated manually using the formula described in Hedges and Olkin^33^ and Jacobson and Truax^34^, respectively.

## Results

### Age trajectory across the lifespan

**Figure 1:**
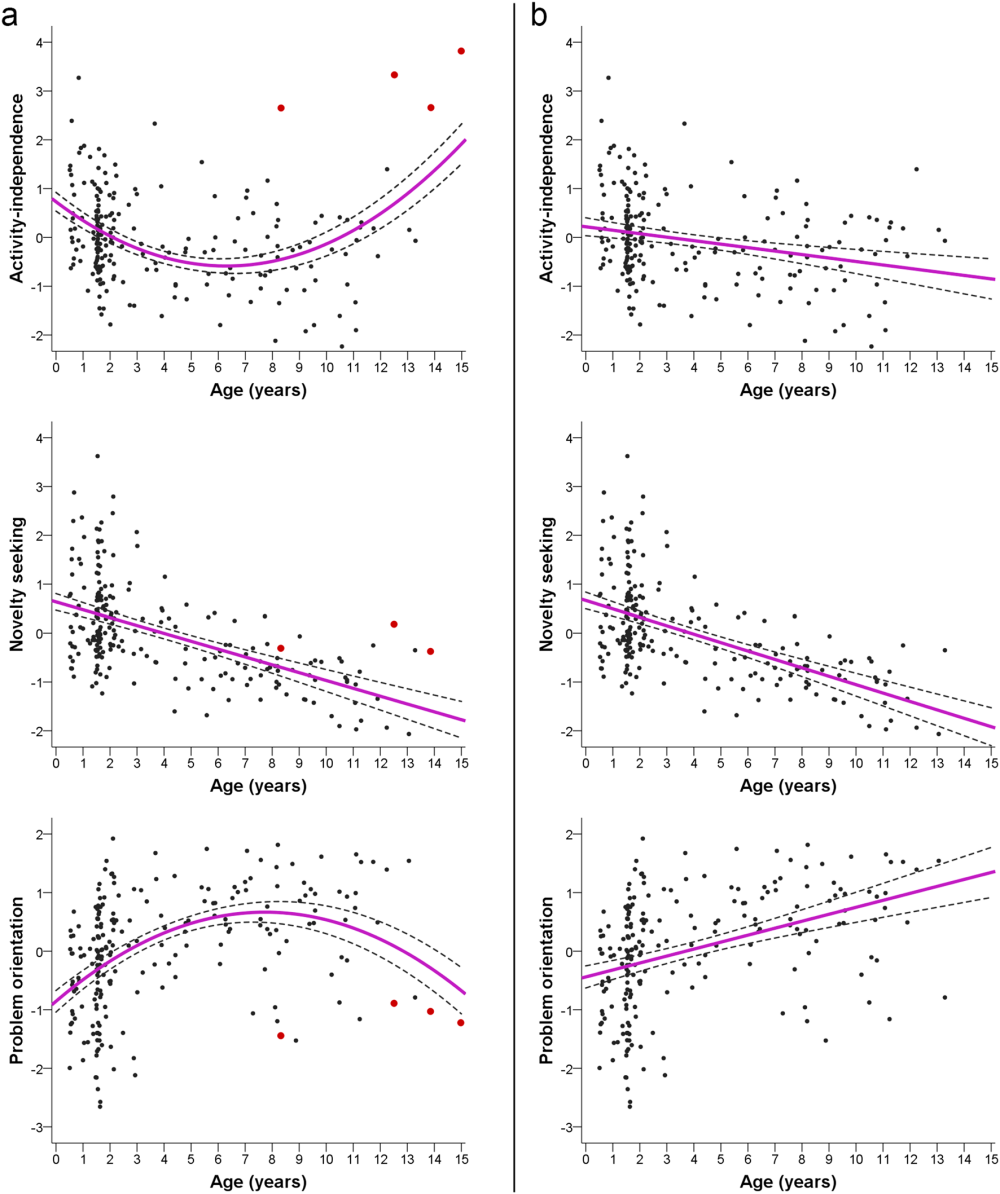
Relationships between age and three personality trait scores of the dogs. (**a**) Relationship on the full sample, the four outlier aged dogs are marked with red dots. (**b**) Relationship after excluding the four outlier aged dogs. Dashed lines represent the 95% confidence intervals of the mean.

We found no linear or quadratic relationship between age and Sociability-obedience (Table 1, Supplementary Fig. S1). For Activity-independence, we found a quadratic relationship with age: young and old dogs were more active-independent than middle-aged dogs (Fig. 1a). For Novelty seeking, both the linear and quadratic regressions were significant and at a similar level of strength. However, as the scatter plot of individual trait scores vs. age showed a linear decrease with age in the trait score with a small increase at geriatric age (> 10  years), we accepted the linear version (Fig. 1a). For Problem orientation both the linear and quadratic functions were significant, though the relationship was stronger in the case of the quadratic function; young and old dogs seem to be less focused on problems than middle-aged dogs (Fig. 1a). Finally, a weak linear increase was found in the case of Frustration tolerance (Supplementary Fig. S1).

### Impaired dogs

The box plots of the five personality traits of dogs aged > 8 years identified one negative outlier value in the case of the Sociability-obedience trait and four positive outliers in the Activity-independence trait (one of these four belonged to the same dog with the extreme negative Sociability-obedience score; see Table 2). No outliers were found for the remaining three traits. Compared to average aged dogs, the four outlier dogs were extremely active, more interested in novel or distracting stimuli, and less successful in solving problems (Table 2).

**Table 2:** (**a**) Means, standard deviations and percentiles of the personality trait scores (z scores) of the aged dogs (> 8 years) without the four outliers (N = 36), and (**b**) the individual scores of the four outlier dogs (extreme values are in boldface).

Percentile	Sociability-obedience	Activity-independence	Novelty seeking	Problem orientation	Frustration tolerance
**a)**					
Mean	0.016	− 0.464	− 1.032	0.549	0.323
SD	0.856	0.883	0.517	0.925	1.055
Percentile 25	− 0.454	− 1.079	− 1.364	− 0.068	− 0.194
Percentile 50	0.053	− 0.254	− 0.959	0.799	0.816
Percentile 75	0.785	0.215	− 0.612	1.281	1.010
**b**)					
*Dog ID *(*age*)					
Dog51 (8.32 years)	0.721	**2.652**	− 0.309	− 1.443	− 0.796
Dog132 (12.51 years)	0.065	**3.331**	0.183	− 0.891	0.493
Dog141 (13.86 years)	− **3.612**	**2.660**	− 0.374	− 1.029	0.664
Dog174 (14.98 years)	− 0.754	**3.819**		− 1.222	0.123

In Novelty seeking one outlier dog had a missing value because the owner did not wish to participate in the Novel object test as he deemed it too stressful for his dog.

These four dogs affected the general age trajectory of two of our traits: after their exclusion, the age trajectories of Activity-independence and Problem orientation were better described by linear functions instead of quadratic ones, the former showing a negative relationship with age, the latter a positive (Table 1, Fig. 1b). The results for the other three traits did not change markedly.

### Mean-level change

**Figure 2:**
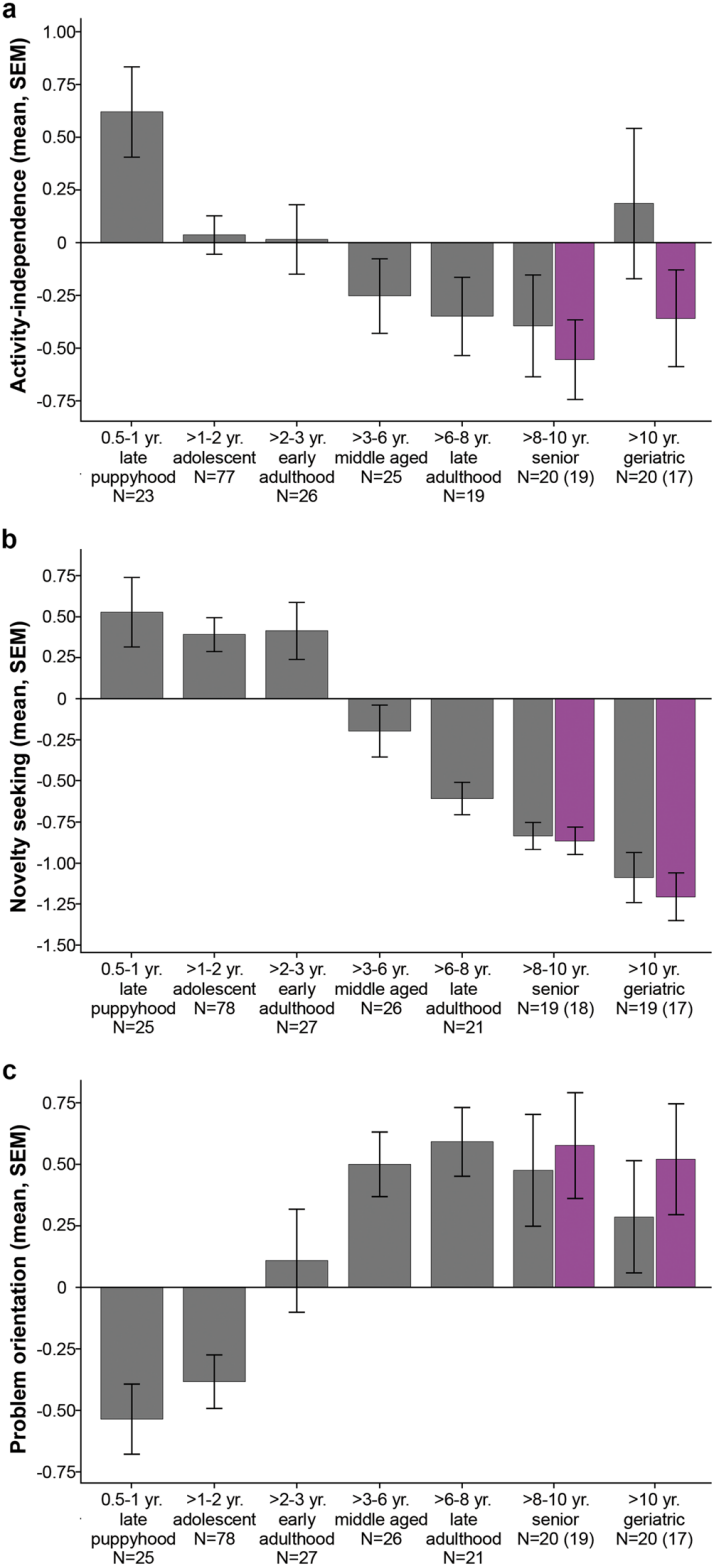
Differences between seven age groups in (**a**) Activity-independence, (**b**) Novelty seeking, and (**c**) Problem orientation traits. The grey bars in the senior and geriatric groups represent the full sample, the lilac bars the sample after excluding the four outlier dogs.

In the cases of Sociability-obedience and Frustration tolerance no significant difference between the seven age groups were found (F_6,208_ = 0.694, *p* = 0.654; F_6,181_ = 1.587, *p* = 0.153, respectively). Mean-level changes for these traits can be found in Supplementary Table S3. For Activity-independence we found a significant difference between the age groups (F_6,203_ = 2.942, *p* = 0.009). The score of the groups decreased continuously but moderately until the end of senior age, and only dogs in late puppyhood (0.5–1  years) differed from dogs in late adulthood (> 6–8 years, *p* = 0.035) and senior age (> 8–10 years, *p* = 0.007) (Fig. 2a). The largest decrease (mean-level change: 68% of the SD) occurred between the puppies and adolescent (> 1–2 years) dogs; at later ages the rate of change was much smaller (Table 3). The quadratic relationship in this trait was largely due to an increase from senior to geriatric (> 10 years) age, and it was primarily caused by the four outlier dogs. Consequently, after excluding these dogs, we found more differences between the groups (F_6,199_ = 4.543, *p* < 0.001), with dogs in late puppyhood being more active-independent than dogs in middle age (> 3–6 years), late adulthood, senior age and geriatric age (*p* < 0.026 for all), and adolescent dogs more active than senior dogs (*p* = 0.049). Also, after excluding the outliers, the life-long mean-level change from late puppyhood to geriatric age became three times larger (98% of the SD) than on the full sample (Table 3).

**Table 3:** Descriptive statistics of the seven age groups in Activity-independence, Novelty seeking, and Problem orientation traits.

	Late puppyhood (0.5–1 years)	Adolescent (> 1–2 years)	Early adult (> 2–3 years)	Middle age (> 3–6 years)	Late adult (> 6–8 years)	Senior (> 8–10 years)	Geriatric (> 10 years)	Life-long change
**Activity-independence**								
N	23	77	26	25	19	20 (*19*)	20 (*17*)	–
mean (M)	0.618	0.035	0.014	− 0.254	− 0.351	− 0.396 (− *0.557*)	0.184 (− *0.360*)	–
SD	1.033	0.803	0.847	0.890	0.817	1.082 (*0.832*)	1.602 (*0.951*)	–
M2 − M1		− 0.583	− 0.021	− 0.269	− 0.097	− 0.045 (− *0.206*)	0.580 (*0.196*)	− 0.434 (− *0.979*)
Hedges' g		− 0.679	− 0.025	− 0.309	− 0.112	− 0.047 (− *0.249*)	0.425 (*0.221*)	− 0.327 (− *0.980*)
**Novelty seeking**								
N	25	78	27	26	21	19 (*18*)	19 (*17*)	–
mean	0.527	0.389	0.412	− 0.200	− 0.609	− 0.837 (− *0.866*)	− 1.091 (− *1.208*)	–
SD	1.074	0.919	0.915	0.815	0.449	0.370 (*0.358*)	0.678 (*0.608*)	–
M2 − M1		− 0.138	0.023	− 0.612	− 0.410	− 0.228 (− *0.257*)	− 0.254 (− *0.342*)	− 1.617 (− *1.734*)
Hedges' g		− 0.144	0.025	− 0.705	− 0.605	− 0.550 (− *0.627*)	− 0.465 (− *0.690*)	− 1.748 (− *1.893*)
**Problem orientation**								
N	25	78	27	26	21	20 (*19*)	20 (*17*)	−
mean	− 0.537	− 0.385	0.107	0.499	0.590	0.475 (*0.575*)	0.285 (*0.520*)	−
SD	0.722	0.975	1.094	0.680	0.650	1.022 (*0.942*)	1.034 (*0.935*)	−
M2 − M1		0.153	0.492	0.392	0.091	− 0.116 (− *0.015*)	− 0.189 (− *0.055*)	0.823 (*1.058*)
Hedges' g		0.166	0.489	0.428	0.137	− 0.136 (− *0.018*)	− 0.184 (− *0.059*)	0.942 (*1.300*)

The mean difference (M2 − M1 = mean of the younger group subtracted from the mean of the older group), and standardised mean difference (Hedges’ g) is calculated from each age group to the next, as well as from late puppyhood to geriatric age (life-long change). In the senior, geriatric, and the life-long change columns, the numbers in brackets represent the values after excluding the four outlier dogs.

Regarding age group differences in Novelty seeking (Chi^2^ = 80.611, *p* < 0.001), the group scores were at the same level until ~ 3 years of age, then continuously decreased (Fig. 2b). Accordingly, the youngest three groups (late puppyhood, adolescence and early adulthood) did not differ from each other (*p* > 0.854 for all), but all three groups received higher scores than dogs in late adulthood, senior, and geriatric age (*p* < 0.001 for all), and middle-aged dogs also received higher scores than geriatric dogs (*p* = 0.033). The mean-level change was negligible between late puppyhood and early adulthood, jumped to ~ 70% of the SD in middle age, then the magnitude of change decreased slowly from each age period to the next (Table 3). The life-long mean-level change from late puppyhood to geriatric age was 174.8% of the SD. Excluding the four outlier dogs did not cause marked changes in these results.

Regarding age group differences in Problem orientation (F_6,210_ = 8.425, *p* < 0.001), the group scores increased continuously from late puppyhood until late adulthood (from 0.5 to 8 years of age), then showed a small decrease in senior and geriatric groups (Fig. 2c). Accordingly, dogs in late puppyhood and adolescence received lower scores than middle-aged, late adult, senior, and geriatric dogs (*p* < 0.036 for all). The mean-level change was the largest (43–49% of the SD) from adolescents to middle age, then became negligible (Table 3). The life-long mean-level change from late puppyhood to geriatric age was 94.2% of the SD. After excluding the four outlier dogs, the trait score no longer decreased in the senior and geriatric groups and the life-long mean-level change became higher (130% of the SD) than on the full sample.

### Individual differences in personality change

On the longitudinal sample (Table 4), Sociability-obedience did not differ significantly between test and re-test at the group level, but five individuals changed significantly (two increased, three decreased). Activity-independence significantly decreased from Test 1 to Test 2, and eight individuals decreased significantly, in harmony with the general age trajectory of this trait. Novelty seeking also decreased from Test 1 to Test 2, but only three dogs decreased significantly, whereas one dog increased significantly, which partly contradicts the general decrease with age we observed in the cross-sectional sample. Problem orientation increased from Test 1 to Test 2, and five individuals increased significantly, in accordance with the result of the age trajectory of this trait. Frustration tolerance did not differ significantly between the two test sessions, and only two dogs changed significantly (both increased).

**Table 4:** Changes in five personality traits between the two test sessions (analysed with paired t-test), and distribution of the individual dogs based on their Reliable Change Index (RCI) scores.

Personality trait	Mean T1	Mean T2	t	*p*	Increase (N (%) of dogs)	Decrease (N (%) of dogs)	No sig. change (N (%) of dogs)
Sociability-obedience	− 0.084	− 0.119	0.353	0.726	2 (5.4%)	3 (8.1%)	32 (86.5%)
Activity-independence	0.065	− 0.198	4.288	< 0.001	0	8 (22.2%)	28 (77.8%)
Novelty seeking	0.044	− 0.176	2.398	0.022	1 (2.7%)	3 (8.1%)	33 (89.2%)
Problem orientation	0.006	0.522	5.490	< 0.001	5 (13.5%)	0	32 (86.5%)
Frustration tolerance	0.000	0.170	1.595	0.122	2 (7.1%)	0	26 (92.9%)

The distribution expected by chance would be 2.5% of the dogs to significantly increase, 2.5% to decrease, and 95% to show no significant change from Test 1 to Test 2^2^.

**Figure 3:**
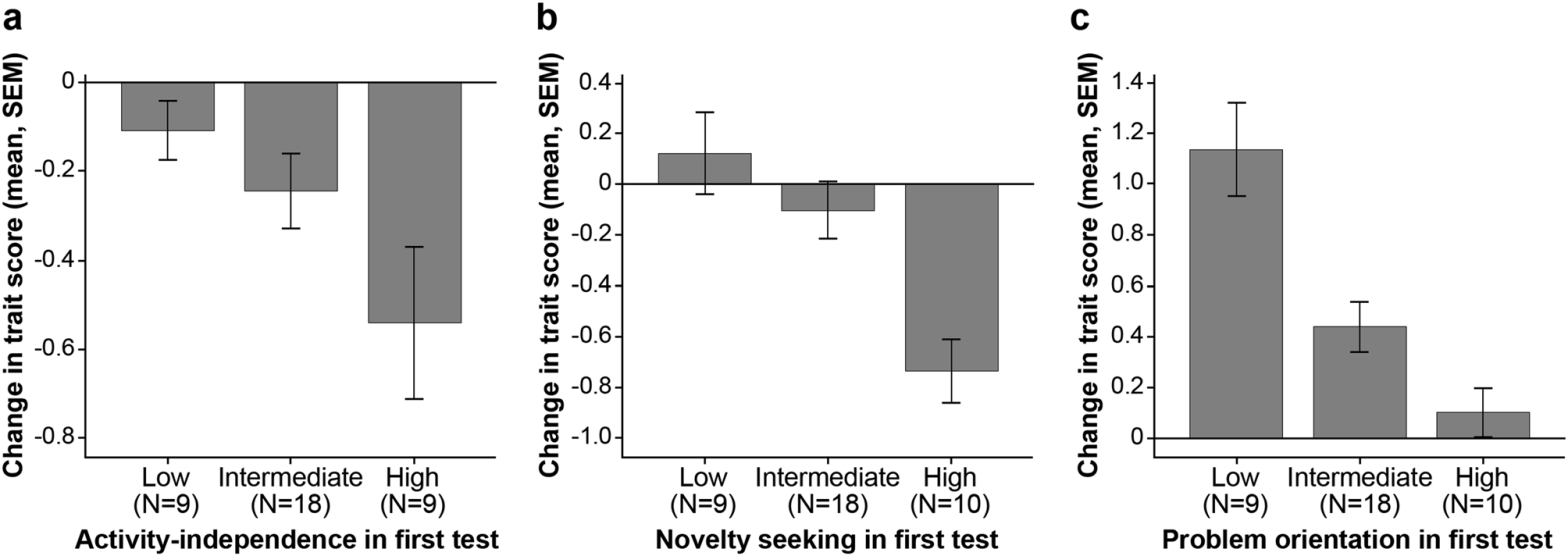
Difference in the magnitude of change in the (**a**) Activity-independence, (**b**) Novelty seeking, and (**c**) Problem orientation traits between the three dog groups created based on the dogs’ scores in the first test. A positive score indicates an increase from Test 1 to Test 2.

To test if individuals with more ‘mature’ personality profile change more/less than others we divided the dogs into three groups according to their performance in the first test session: Low (1st quartile), Intermediate (2nd, 3rd quartile), and High (4th quartile). No significant differences between these groups were found in Activity-independence (F_2,33_ = 3.214, *p* = 0.053), although dogs with low trait scores in Test 1 tended to decrease less than dogs with high scores (*p* = 0.050; Fig. 3a). In Novelty seeking (F_2,34_ = 9.246, *p* < 0.001), the dogs with high scores in Test 1 decreased more than the other two groups (High vs. Low: *p* < 0.001; High vs. Intermediate: *p* = 0.004; Fig. 3b). In Problem orientation (F_2,34_ = 13.936, *p* < 0.001), dogs with low scores in Test 1 increased more than the other two groups (Low vs. Intermediate: *p* = 0.001; Low vs. High: *p* < 0.001; Fig. 3c).

## Discussion

The main objective of this study was to investigate the trajectory and dynamics of personality changes over the majority of the dogs’ life course. In general, we expected that, in accordance with the cumulative continuity principle^3–5^, personality change would most prominently occur until the end of middle age (~ 6 years of age) and the rate of change would slow down in the senior and geriatric ages. This expectation was confirmed regarding the Activity-independence and Problem orientation traits: the former decreased, the later increased with age following this pattern. The age group comparisons revealed that the most pronounced decrease in Activity-independence occurred between late puppyhood and adolescence (~ 1 year of age), while Problem orientation increased mostly during adolescence and early adulthood (~ 1–3 years of age). Not only the age dynamics but also the magnitude of change in these traits corresponds well with human data. The cumulative personality change across the human lifespan is estimated to be around 1 SD regarding the neuroticism, agreeableness, and conscientiousness traits^2, 4^, which fits well with our findings regarding Activity-independence (~ 98% SD) and Problem orientation (130% SD). Contrary to these two traits, the results regarding Novelty seeking failed to support our hypothesis, as this trait started to decline only in middle age. Still, Novelty seeking showed the strongest relationship with age and the life-long change in this trait (~ 1.8 SD) is very high by human psychological standards^2^.

The general direction of age-related changes in all three traits is consistent with the dog literature. In most studies, activity and related traits were found to decrease in older dogs ^17, 20, 23, 35–37^, and this decrease is generally attributed to a natural decline in sensory and motor systems^30^. While we also showed that activity continuously decreases with age, we also showed that the decrease is most pronounced at a younger age when biological ageing has not yet progressed. This suggests that other factors are (also) in play, such as a sexual maturation, or the dwindling interest/motivation to explore familiar surroundings^38, 39^. Dogs’ attentiveness and ability to solve problems had also been shown to improve in adolescence and early adulthood^22, 37, 40^, in harmony with our results. However, studies also showed that cognitive functions, including problem solving abilities, decline in old age^41^, in contrast to which we found only a small decrease in geriatric age in Problem orientation. It is possible that our two problem solving tasks were not difficult enough to reveal declining performance in the older age. Finally, similar to our results, traits related to the dogs’ response to novelty were also shown to decrease in older dogs^22, 24, 42^. Higher interest in novelty, as well as a lower ability to focus on problems in younger dogs could be due to their greater levels of distractibility^22, 37^ or less well developed behavioural control^43^. As dogs’ age and accumulate experiences, they become desensitised and less reactive to novel stimuli, such as moving objects, compared to young animals that are still learning and exploring^24^. The attentional control of dogs is also likely to improve with age as they learn (and are trained) to inhibit their immediate behavioural responses towards interesting or distracting stimuli.

For the Sociability-obedience trait, we found no linear or quadratic relationship with age, no significant difference between the seven age groups, nor any difference between the two test sessions on the longitudinal sample. Contrary to these results, other studies reported sociability-related traits to decrease in older age^18, 19, 35^. While we observed no general age trends on the population level, we found five individuals changed significantly between the two test sessions. This suggests that the Sociability-obedience trait is less influenced by biological or environmental factors associated with ageing, but it is subject to individual factors. Regarding Frustration tolerance, only a weak linear relationship was found with age, with older dogs being slightly more tolerant to frustrating situations (i.e., unreachable food) than younger dogs. However, no significant difference was found between the seven age groups, nor any difference between the two test sessions on the longitudinal sample. Thus, dogs may become less easily agitated (or less likely to show it), or they may became less motivated in trying to obtain the (inaccessible) rewards as they age, but this trend seems to be a small and gradual change and/or is the result of a few individuals changing markedly over time.

Note that while the cross-sectional and longitudinal analyses agreed in four out of five traits, the results of the longitudinal assessment did not fully confirm the cross-sectional results of Novelty seeking. While all dogs in the longitudinal sample either reached middle-age in their second test or were middle-aged/older during their first test, only 4 individuals changed significantly in the course of ~ 4 years, and the score of one of them even increased. The equipment we used in the tests related to this trait were the same in both test sessions, and this familiarity could have lead to divergent changes, depending on the dogs’ initial attitude. Dogs that were highly interested in these objects in Test 1 lost interest in the no longer ‘novel’ stimuli for the second test, thus their trait score decreased. In dogs that were rather reserved or cautious in Test 1, familiarity with these stimuli led to higher interest and increased trait score in the second test. Svartberg et al.^26^ also reported an increase in Curiosity/Fearlessness (reaction to potentially frightening novel stimuli) from the first to the second test session, due to fearful dogs becoming more confident when exposed to the same objects and situations the second time. Contrary to Svartberg et al., we did not find a significant increase in our Novelty Seeking factor score over multiple tests probably because the VIDOPET only included one fear-evoking test situation, in contrast to the ‘Dog Mentality Assessment’ used by Svartberg et al., which had several (such as the gun shot, sudden loud noise, ghost). Thus our Novelty seeking factor corresponds mainly to the Curiosity part of the Curiosity/Fearlessness factor. Still, the different cross-sectional and longitudinal results regarding Novelty seeking calls attention to the fact that longitudinal designs also have confounding effects, such as familiarity with the test stimuli.

Our results also showed that the age trajectories of Activity-independence and Problem orientation (but not Novelty seeking) were affected by four aged dogs with outlying scores. After excluding these dogs, the increase in Activity-independence and a decline in Problem orientation in the geriatric age cohort disappeared, leading to stronger linear age associations. While the quadratic regression curves also remained significant in both traits, they reflected only a change in the steepness of the slope, and no longer implied a significant change in the direction of association (see similar results in^23, 44^). Although we lacked specific diagnostic tests, the extremely high activity level, combined with lower problem solving skills indicates potential age-related impairments in these dogs. Previous studies have shown that cognitively impaired aged dogs are characterised by hyperactivity (typically showing undirected, stereotypical behavioural patterns) and activity levels similar to young dogs^38, 42^. Thus, a sudden increase in activity in old age could be an indicator of possible sensory impairments leading to higher anxiety/disorientation in a novel environment^42, 45^, or even indicate neurodegenerative changes associated with behavioural control mechanisms, which disrupt the normal decline^24^.

In addition to these four outlier dogs, we also found others that deviated from the general age trajectory reflected in significant individual differences in personality change in the longitudinal sample. In accordance with our expectation based on human studies^3, 13^, dogs with a more ‘mature’ personality profile in Test 1 (i.e., lower Activity-independence and Novelty seeking and higher Problem orientation) changed less than the others. Although we cannot rule out the possibility that these differences reflect simple floor/ceiling effects, that is, these dogs changed less because they had less room to change, we need to note that none of the dogs in our sample reached the hypothetical minimum or maximum of any of the traits.

Taken together, the analyses conducted here fill important gaps in our knowledge and can help in determining what can be considered normal behavioural change during ageing regarding both the direction and magnitude. Our results showed that changes in personality occur unevenly during the dogs’ life course and individuals differ significantly in personality development, which raises caution against over-generalisation of the global age trends. Moreover, the fact that only four outlier dogs (2% of the sample) significantly affected the general age trajectory of some traits, highlights the importance of the composition of the (aged) population involved in future study samples. However, we also acknowledge that investigating only one breed (Border collie) strongly limits the generalisability of our results as studies have found a large (almost two-fold) divergence in the longevity between different breeds^46^, which could reflect different ageing dynamics^47^. Although by assessing only one breed we were able to avoid such bias, similar research should be replicated in other breeds. In addition, although the VIDOPET attempted to measure a broad range of personality traits in pet dogs, it was not suitable to detect individual variability in two common personality traits, fearfulness and aggression. Due to ethical and safety reasons only one subtest addressed the dogs’ fearfulness and propensity to display aggression, and we also excluded overly shy dogs from the sample. If age-related changes in these particular traits are of interest in future studies, it may be necessary to include additional subtests, as well as to explore a more diverse sample of pet dogs.

## Supplementary information

Supplementary Information.

## Data Availability

The datasets analysed during the current study are available in the Figshare repository, https://doi.org/10.6084/m9.figshare.5477932.v2 (factor_scores and test_retrest data sheets).
