# Supplementary material for: Individual and group level personality change across the lifespan in dogs
Source: Sci Rep. 2020 Oct 14;10:17276. doi: 10.1038/s41598-020-74310-7 (PMC7560605; doi:10.1038/s41598-020-74310-7)
Supplement: Supplementary file 1 — Supplementary Information. [file 41598_2020_74310_MOESM1_ESM.pdf]

Individual and group level personality change across the lifespan in dogs

Borbála Turcsán, Lisa Wallis, Judit Berczik, Enikő Kubinyi, Friederike Range, Zsófia Virányi

Supplementary Methods. Detailed protocol of the behaviour test (VIDOPET) and definition of the variables coded.

The tests took place indoors, the equipment in the rooms included: a big cardboard box filled with cardboard scrapings, an old t-shirt, an opened umbrella, a water bowl, a bag filled with magazines and a tennis ball, and a large plastic bag, as well as a small table (used for storing some smaller items needed for the test), and a chair. Eight pictures of dog faces were displayed on the four walls 1.5 m above the ground.
The battery took ~ 1 hour to complete, and consisted of 15 subtests carried out in the same order for all dogs, with a short break (5-10 min) after the 7th subtest. The owners were informed about the procedure of each subtest before the onset. Shortening or skipping a subtest was allowed if 1) the owner did not wish his/her dog to participate in that situation for any reason, 2) the dog displayed any behaviours indicating strong fear or aggression (e.g., excessive vocalization, severe growling, shivering, cowering, urination, attempting to escape, snapping, attempting to bite, etc.).

1. Exploration (previously described in[1])

The owner (O) entered the room with the dog on leash. They went to the middle of the room where the O took the leash off and let the dog go (by using a release command if it was necessary). Thus, the dog could explore the room and the objects on the floor while the O stood in the middle of the room ignoring the dog. After 1 min. the experimenter (E) (outside the room) played a previously agreed ring tone which indicated the start of the next test.
Coded variables:
1) Duration of locomotion: any movement of the legs leading to a forward or backward motion
2) Duration of exploration: the dog’s nose is within 10 cm of the floor or any other surface (e.g., wall, table, objects) or dog has placed both front paws on an elevated surface (e.g., window sill, table). Does not include drinking or lying down
3) Duration of being close to O: the dog’s head is within 1m from O
4) Duration of looking at O: the dog's head is oriented towards the O (both when the dog is stationary and when moving).
5) Duration of inactivity: the dog is standing, sitting, or lying, without exploring according to the above definition; also included: transition from standing to sitting or from sitting to lying and scratching.

2. Picture viewing (previously described in[1])

After hearing the ring tone the O went to the table, where he/she found a clipboard with a sheet listing eight different emotions, corresponding to the eight pictures of dog faces on the wall. The O’s task was to walk slowly around the room and note down which picture corresponds to which emotion on the list. The O was requested to spend approximately 5-6 sec in front of each picture, then go to the next one while completely ignoring the dog. The dog was free to move. After 1 min. the E (outside the room) played the same ring tone again. The O then went to the middle of the room, called the dog, and put the leash on him/her.

Coded variables:
1) Duration of moving independently from O: locomotion, only when not in the direction of the O, and not looking at O
2) Duration of exploration: the dog's nose is within 10 cm of the floor or any other surface (e.g., wall, table, objects) or dog has placed both front paws on an elevated surface (e.g., window sill, table). Does not include drinking or lying down
3) Duration of being close to O: the dog's head is within 1m from O
4) Duration of looking at O / following O: the dog's head is oriented towards the O (both when the dog is stationary and when moving) or dog is following the moving O (moving while looking at O or walking behind/ next to O in the same direction as O).
5) Duration of inactivity: the dog is standing, sitting, or lying, without exploring according to the above definition; also included: transition from standing to sitting or from sitting to lying and scratching.

3. Greeting the experimenter (previously described in[1])

The E entered the room, approached the dog-owner pair, and said "Hello" to them. E stopped in front of the dog (just outside her/his reach).

- If the dog approached E in a friendly (tail wagging) or neutral (no tail wagging, no aggression) manner, E petted the dog while talking to it continuously. Then E stepped 1 m away from the dog within reach of the leash. If the dog followed, E petted the dog for the second time. If the dog did not follow, E called the dog then crouched and called the dog again. If the dog still did not go to E, she went to the dog and petted it again.
- If the dog did not approach or showed avoidance, E crouched down and tried to call the dog. If the dog approached E in a non-aggressive manner, E followed the instructions above. If the dog did not show any reaction, E talked to the dog for a while but did not try to get direct contact with the dog anymore then terminated the test.
- If the dog showed any sign of aggression (such as growling, barking), then E remained outside its reach and talked continuously to the dog in a friendly manner for 10 sec. If the dog approached E in a non-aggressive manner, E followed the instructions above. If not, she terminated the test.

The O was instructed to stand still, hold the leash, and not to talk to the dog during the test. The testing continued only if the E could interact with the dog (i.e., pet at least once) during this test, otherwise she terminated the test.
Coded variables:
1) Approaching E (score): when E stops in front of the dog, the dog 0: does not approach the E; 1: approaches hesitatingly or after a while; 2: approaches immediately when called; 3: approaches immediately without calling
2) Greeting E (score): when E stops in front of the dog, the dog 0: shows avoidance, or steps towards E but then stops before reaching her; 1: approaches E but then behaves passively and does not initiate interaction; 2: is friendly, wags its tail, may cuddle up, jump or lick; 3: is very excited/ enthusiastic, with intensive searching for contact and tail wagging
3) Tail wagging (score): when E stops in front of the dog, the dog 0 shows no or very little tail wagging; 1: wags its tail intermittently; 2: wags its tail most of the time

4. Food choice (previously described in[1])

Phase 0: Warm-up

E put a plastic plate (with a piece of food on it) in front of the dog and the dog was allowed to eat it (the owner could encourage the dog if necessary).

Phase 1: Free choice phase (6 trials)
The O was instructed to sit on a chair, fill a questionnaire in and ignore the dog during the phase. The dog was tied with a 1 m long leash to a handle on the floor, while E prepared two similar plates for the test. E, turning her back to the dog, put one piece of sausage on one plate and left the other plate empty. She then put the two plates on the floor 1m apart from each other and 1.5 m in front of the dog (whilst still standing with her back to the dog), then she turned and walked behind the dog. E waited until the dog had clearly looked at both plates (ca. 5 sec), then she released the dog to go to the plates (giving a release command, if necessary). If the dog did not move for 5 sec, the encouragement or the release command was repeated (this time also pushing the dog slightly on the back). After the dog made a choice (nose < 10 cm from the plate), E removed the non-chosen plate.
The trial was repeated six times; the food was placed on a different plate in every consecutive trial, the starting side of the food was counterbalanced among dogs.

Phase 2: Preference phase (6 trials)
The protocol of this phase was similar to the Free choice phase, except that right before the dog was released to make a choice, the O went to the empty plate, crouched down next to it, and pretended as if was really interesting and delicious (e.g. saying "mmm, that is yummy!"). After that the O returned to the chair and continued filling the questionnaire in, without looking at the dog or the plates. Then E released the dog to make a choice. This trial was also repeated six times; again, the position of the food was changed in every trial.
Coded variable:
1) Change in bias: % correct choices (plate with food) in Phase 2 subtracted from % correct choices in Phase 1.

5. Frustration test (previously described in[1])
E tied the dog with a 1 m long leash to a hook (the hook was fixed on the wall in the first session, and on the floor in the second test session), and asked the O to sit on the chair next to the dog and not to interact with the dog during the test. As a warm-up, E held a small piece of sausage in her hand, and moved it in front of the dog from left to right 3 times, then threw it next to the dog (the dog was allowed to eat it). This procedure was repeated in the other direction.
During the test, E stood in front of the dog, turned sideways and did not look at the dog directly. She swung a large piece of sausage on a string in front of the dog's nose (the dog was not quite able to reach it), from left to right for 1 min. When the time was over, E gave a piece of food reward to the dog to resolve the situation.
Coded variables:
1) Duration of orientation to food: the dog's head is facing in the direction of the object or E's hand (swinging the sausage)
2) Duration of looking at E: the dog's head is facing in the direction of the E's face

3) Frequency of snapping: snapping at the food, grabbing with the mouth into the air (may or may not be accompanied by lunging)
4) Frequency of frustration/stress signals: any type of vocalization, yawning, lip licking, stretching, scratching/ grooming itself, or shaking

6. Separation (previously described in[1])

The dog was unleashed and allowed to move freely. The O and E left the room at the same time using different doors without saying anything to the dog. The dog was alone in the room for 1 min. The O and E watched the dog from outside the room, and the separation period was cut short if the dog expressed an excessive stress reaction.

Coded variables:

1) Duration of being close to the doors: at least one foot of the dog is within 1 m of one of the exit doors (marked by a circle on the floor)
2) Duration of looking at the door: the dog's head is oriented towards one of the doors while the dog is stationary (sitting, lying, standing). Not coded when dog is within 1m to the doors
3) Duration of locomotion: any movement of the legs leading to a forward or backward motion
4) Duration of exploration: the dog's nose is within 10 cm of the floor or any other surface (e.g., wall, table, objects) or dog has placed both front paws on an elevated surface (e.g., window sill, table). Does not include drinking or lying down
5) Duration of inactivity: the dog is standing, sitting, or lying, without exploring according to the above definition; also included: transition from standing to sitting or from sitting to lying and scratching.
6) Frequency of frustration/stress signals: any type of vocalization, yawning, lip licking, stretching, scratching/ groom itself, shaking, jumping at the door, or scratching the door

7. Greeting after separation (previously described in[1])

After 1 min. separation, E entered the room (using the same door she left from), and stood right next to the door for 5 sec. If the dog did not approach her during this time, E called the dog and greeted it (following the protocol of Subtest 3 (Greeting the experimenter test, see above). After the greeting, E picked up a toy rope from the windowsill and initiated a tug-of-war play with the dog for 30 sec. Then E put the toy back to the windowsill and left the room without saying anything. Outside, E waited for 5 sec and then instructed the O to enter the room (using the same door he/she left from). The O repeated the same procedure as the E (stopped next to the door, greeted the dog, and then played with it).

Coded variables:

1) Latency of approaching E: from E's entry until the dog is within 20 cm of E
2) Approaching E (score): while E is standing next to door, the dog 0: does not approach the E; 1: approaches hesitatingly or after a while; 2: approaches immediately when called; 3: approaches immediately without calling
3) Greeting E (score): while E is standing next to door, the dog 0: shows avoidance, or steps towards E but then stops before reaching her; 1: approaches E but then behaves passively and does not initiate interaction; 2: is friendly, wags its tail, may cuddle up, jump or lick; 3: is very excited/ enthusiastic, with intensive searching for contact and tail wagging
4) Tail wagging (score): while E is standing next to door, the dog 0 shows no or very little tail wagging; 1: wags its tail intermittently; 2: wags its tail most of the time

5) Play intensity (score): when the E invites the dog to play, the dog 0: does not play; 1: mouths the toy sometimes, but no real play or needs some encouragement; 2: mouths the toy and sometimes pulls it/brings it back; 3: is very enthusiastic, plays more than 90% of the time
6) Latency of approaching O: from O's entry until the dog is within 20 cm of O
7) Approaching O (score): while O is standing next to door, the dog 0: does not approach the O; 1: approaches hesitatingly or after a while; 2: approaches immediately when called; 3: approaches immediately without calling
8) Greeting O (score): while O is standing next to door, the dog 0: shows avoidance, or steps towards O but then stops before reaching her; 1: approaches O but then behaves passively and does not initiate interaction; 2: is friendly, wags its tail, may cuddle up, jump or lick; 3: is very excited/ enthusiastic, with intensive searching for contact and tail wagging
9) Tail wagging (score): while O is standing next to door, the dog 0 shows no or very little tail wagging; 1: wags its tail intermittently; 2: wags its tail most of the time

Between tests 7 and 8 a break of 5-10 minutes was taken during which the owner and dog remained outside of the test room.

<u>8. Problem I (cage) (previously described in[1])</u>

Warm-up

E put a 75 x 55 x 60 cm large cage in the middle of the room, fixed it to the floor and let the dog explore it. Then she put a piece food in front of the cage and the dog was allowed to eat it (the owner could encourage the dog if necessary).

Trial 1

The O sat on a chair 1.5 m from the cage and held the dog. E turned her back to the dog and put a piece of food in a small container at the end of a ca. 30 cm long string then placed the string halfway inside the cage. The other end of the string stuck out of the cage. E stepped away from the cage and the O released the dog. The O was instructed to remain sitting but he/she was allowed to encourage the dog verbally or with hand cues (e.g. pointing at the direction of the cage). The test was terminated a) if the dog pulled out the string and got the food; b) if the dog did not interact with the cage for 1 min.; c) or after 5 min. If the dog was not able to get the food, E gave it to the dog and skipped Trial 2.

Trial 2 (blocked trial)

If the dog had successfully pulled out the string in Trial 1, E, turning her back to the dog, put another piece of food on the string. She again placed the string halfway in the cage but this time she fixed the string to the cage by a hook, so the dog was not able to pull it out. Then E stepped away from the cage and the O released the dog. The O was allowed to encourage the dog verbally but only in the first 30 sec of the test. The test was terminated a) if the dog did not interact with the cage for 1 min.; b) or after 5 min. At the end, E gave the food to the dog.

Coded variables:

1) Latency of success: in Trial 1, from releasing the dog until the strap has been pulled out of the cage completely or the dog has otherwise obtained the sausage
2) Duration of orientation to cage: in Trial 2, the dog's nose or paw is within 10cm of the cage and the dog shows an interest in the cage (e.g. sniffing, trying to get the food, walking around the cage while

oriented towards it). Does not include looking at the cage passively, even when physical contact with the cage is made
3) Duration of looking at E or O: in Trial 2, the dog's head is oriented towards O or E
4) Duration of being close to O: in Trial 2, the dog's head is within 1m from O
5) Frequency of vocalization: in Trial 2, any type of vocalization
6) Latency to give up: in Trial 2, from releasing the dog until the dog stops interacting with the cage for 1min (maximum: 300 sec).

9. T-shirt test (previously described in[1])

The dog was off the leash. The O was instructed to call the dog to him/her then tried to put a T-shirt on the dog (by putting it first over the head, then putting the front legs through the arms and finally knotting the T-shirt at the back). During the procedure, the O was not allowed to talk to the dog (he/she was given a lollipop to prevent him/her to talk), but he/she was allowed to gently hold the dog back if it tried to escape. If the dog still managed to escape, the O could call it back three times. If the dog was not willing to return, or the O could not put the T-shirt on within 1 min. the test was terminated. After the T-shirt had been put on the dog, the O stood up and walked around the room looking at the pictures on the wall (while ignoring the dog). Then the O called the dog and took off the T-shirt and comforted the dog if necessary.

Coded variables:

1) Duration of inactivity: after the t-shirt has been put on, the dog is standing, sitting, or lying, without exploring; also included: transition from standing to sitting or from sitting to lying and scratching.
2) Duration of looking at O: after the t-shirt has been put on, dog's head is oriented towards O while stationary (not coded when the dog is following O)
3) Duration of following O: the dog is following the moving O (moving while looking at O or walking behind/ next to O in the same direction as O)
4) Duration of moving independently from O: locomotion, only when not in the direction of the O, and not looking at O

10. Obedience (previously described in[1])

The O and the dog (off leash) went to the far end of the room facing the doors. E crouched down next to the right side door behind a plastic box filled with crumpled newspaper. The O commanded the dog to “sit”, “lay down” then “stay”, then walked towards the left side door as if to leave. But he/she stopped next to the door, turned back to the dog and stood still for 15 sec, then called the dog to him/her. Meanwhile E was rustling in the box acting as if searching for something. If the dog got distracted by the noise, the O could repeat the commands.

Coded variables:

1) Latency of obey commands: the mean latency to obey the "sit" and "lay down" commands (from the first verbal or non-verbal command until the dog sat (rumps on the floor), or lay down completely (whole body on the floor))
2) Latency of distract: from the sit command until the dog starts to move towards the distraction (even if the O manages to call it back)
3) Latency of recall: from the O’s first call command until the dog is within 10 cm of the O.
4) Mean latency of obey “come”: at six time points during all 15 subtests, the O is asked to recall their off-lead dog. The mean latency to obey the “come” command was calculated by averaging these six

latencies (from the first verbal or non-verbal command until the dog makes the first movement towards the O)
4) Mean recall latency from cage: during the Problem I (cage) test, before and after its Trial 1, the O is asked to recall their off-lead dog. The mean recall latency from cage was calculated by averaging these two latencies (from the first verbal or non-verbal command until the dog makes the first movement towards the O)

11. Threatening approach (previously described in[1])

The O and the dog (on leash) stood at the far side of the test room facing the doors. The O commanded the dog to sit, then stepped one step behind it holding the leash loosely. She/he was asked not to talk to or interact with the dog during the test. E stood between the two doors facing the dog. When the dog looked at her, E started to slowly approach the dog with a slightly bent upper body, staring steadily in the eyes of the dog, and without any verbal communication. If the dog broke eye contact, E tried to get back the dog's attention by making some noise (cough, stamping). The approach was terminated a) if E reached the dog or the dog approached E in a friendly manner (in both cases E petted the dog); b) if the dog reacted aggressively (e.g. excessive growling or barking, snapping or attacking); c) if the dog moved away and hid behind the O.

Coded variables:

1) Latency of aversive reaction: from the first step the E makes until the dog shows any ‘aversive’ (aggressive or avoidant) reaction, like moving away, hiding, barking, or growling.
2) Latency of friendly reaction: from the first step the E makes until the dog shows any ‘friendly’ reaction, like tail wagging, moving towards the E, showing behaviours of appeasement
3) Reaction to threatening (score): the behaviour shown just before the test is terminated 0: friendly, appeasing, i.e. approaching E with tail wagging; 1: passive, i.e. no approach and no avoidance, may look away, and may wag tail intermittently; 2: avoidant, i.e. hid behind the O/ moved away from the E (with low tail and ear position); 3: aggressive, i.e. bark, growl, snap, and/ or lunge towards E
4) Barking (score): the dog barks one or more times while E is approaching: 0: no; 1: yes
5) Growling (score): the dog growls one or more times while E is approaching: 0: no; 1: yes
6) Friendly approach (score): the dog steps towards E in a friendly, appeasing or submissive way while E is approaching: 0: no; 1: yes
7) Moving off (score): while E is approaching the dog 0: does not move away from the approaching E; 1: moves away from the E, but does not move behind O; 2: moves behind O.

12. Post-threat interaction (previously described in[1])

Following the threatening approach, E crouched down and called the dog in a friendly way, following the protocol of the Greeting the experimenter test. This test also served to resolve the Threatening approach and reconnect the experimenter and the dog, and the testing continued only when the dog was relaxed in the presence of the experimenter.

Coded variables:

1) Approaching E (score): when E crouches down the dog 0: does not approach the E; 1: approaches hesitatingly or after a while; 2: approaches immediately when called; 3: approaches immediately without calling, or has already approached her in a friendly way during the threatening episode
2) Greeting E (score): when E crouches down the dog 0: shows avoidance, or steps towards E but then stops before reaching her; 1: approaches E but then behaves passively and does not initiate interaction;

2: is friendly, wags its tail, may cuddle up, jump or lick; 3: is very excited/ enthusiastic, with intensive searching for contact and tail wagging
3) Tail wagging (score): when E crouches down the dog 0 shows no or very little tail wagging; 1: wags its tail intermittently; 2: wags its tail most of the time

13. Problem II (bin) (previously described in[1])

E tied the dog with a 1 m long leash to a hook (the hook was fixed on the wall). The O placed a small bin (31 cm high, 22 cm in diameter) 1.5 m in the front of the dog. The O crouched down behind the bin, called the dog's attention and demonstrated to the dog how to remove the lid of the bin to get a piece of sausage from it (lifting the lid, showing a piece of sausage in the bin, closing the lid, then lifting it again). The O repeated this demonstration four times. After the fourth time, the O put the lid back on the bin, and stepped one step back. E then removed the leash and the O verbally encouraged the dog to manipulate the bin. If the dog was not able to get the food within 1 min., E gave it to the dog.

Coded variables:

1) Duration of orientation to bin: the dog's nose or paw is within 10cm of bin, dog is oriented towards bin
2) Latency of success: from releasing the dog until the cover of the bin has been completely removed

14. Novel object (previously described in[1])

The O and dog (off leash, restrained by the O) positioned themselves at the far end of the room, facing the wall. E placed a 30 x 15 x 10 cm toy dog in the middle of the room, turned it on and then stepped back to the right wall and stood still. The toy, when turned on, started rolling on the floor vigorously and made a noise (resembling human laughter) for 30 sec then stopped. Upon hearing the noise from the toy, O released the dog, then stood still facing the toy for 1 min. The toy had an automatic movement sensor built in, and started a second round of rolling if the dog came close enough to explore it. If the dog did not make the toy turn on a second time, E turned it on again by walking past it across the room to the opposite wall. After 1 min., E turned the toy off manually. If the dog did not approach the toy or seemed to be afraid of the toy, the O showed the turned-off toy to the dog and encouraged the dog to sniff it.

Coded variables:

1) Duration of being close to O: the dog's head is within 1m from O
2) Duration of being close to toy: the dog's head is within 1m from the toy
3) Duration of looking at O: the dog's head is oriented towards O
4) Duration of looking at toy: the dog's head is oriented towards toy
5) Duration of looking at E: the dog's head is oriented towards E

15. Ball play (previously described in[1])

The O and dog went to the far end of the room, while the dog was off-leash. The O threw a tennis ball three times, and encouraged the dog to retrieve it, and give it back to him/her. After the third throwing, the O stopped interacting with the dog, stood still and ignored the dog for 15 sec. Then the O took the ball back, put it on the windowsill and slowly walked around the room looking at the pictures on the wall (while ignoring the dog).

Coded variables:

1) Follow the ball (score): the number of times the dog follows the thrown-away ball
2) Grab the ball (score): the number of times the dog grabs the thrown-away ball
3) Return the ball (score): the number of times the dog returns with the ball to within 1.5m of the O within 5 seconds after first touching ball
4) Give out the ball (score): the number of times the dog gives the ball to the O or drops it within 1.5 m of the O within 5 seconds after first touching the ball
5) Frequency of gaze alternation: after the ball has been placed on the window sill, looking from the ball to the O or from the O to the ball

References

1. Turcsán, B. *et al.* Personality traits in companion dogs—Results from the VIDOPET. *PLoS One* **13**, e0195448 (2018).

Supplementary Figure S1. Relationships between age and two personality trait scores of the dogs. **(a)** Relationship on the full sample, the four outlier aged dogs are marked with red dots. **(b)** Relationship after excluding the four outlier aged dogs. Dashed lines represent the 95% confidence intervals of the mean.

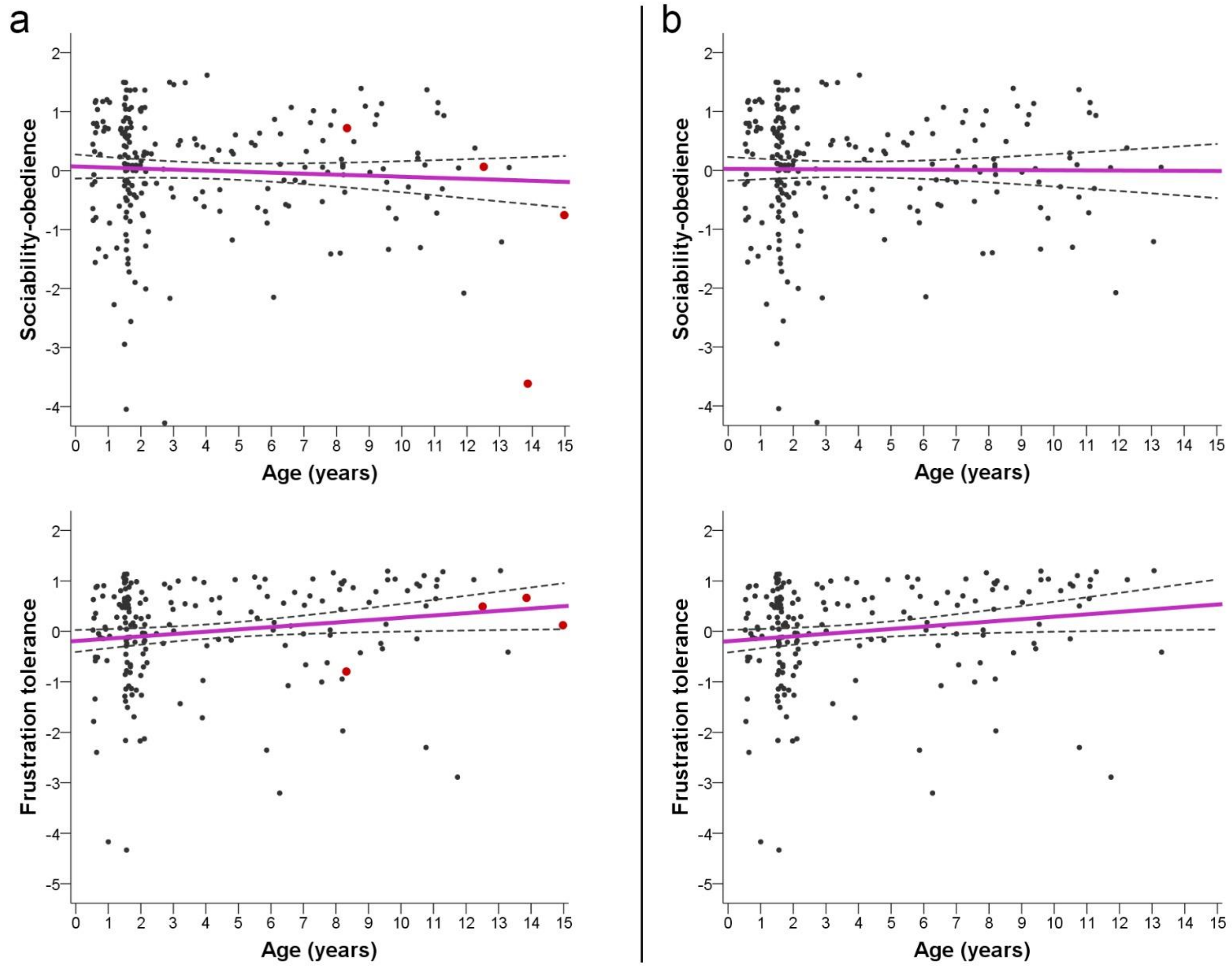

Supplementary Table S1. Distribution (number and percent) of the dogs according to their age groups in the cross-sectional sample, and in the longitudinal sample at first and second testing.

**Age group**	**Cross-sectional sample**	**Longitudinal sample T1**	**Longitudinal sample T2**
Late puppyhood (0.5-1 yr.)	25 (11.52%)	5 (13.51%)	0
Adolescence (>1-2 yr.)	78 (35.94%)	15 (40.54%)	0
Early adulthood (>2-3 yr.)	27 (12.44%)	6 (16.22%)	0
Middle age (>3-6 yr.)	26 (11.98%)	8 (21.62%)	20 (54.05%)
Late adulthood (>6-8 yr.)	21 (9.68%)	3 (8.11%)	7 (18.92%)
Senior age (>8-10 yr.)	20 (9.22%)	0	8 (21.62%)
Geriatric age (>10 yr.)	20 (9.22%)	0	2 (5.41%)

Supplementary Table S2. The five personality traits used in the analyses. E: experimenter; O: owner. Loadings > 0.4 are in boldface.

**Subtest, *Subtest component***	Sociability-obedience	Activity-independence	Novelty seeking	Problem orientation	Frustration tolerance
Greeting the experimenter, *Greeting*	**0.775**	0.151	0.068	-0.087	0.003
Greeting after separation, *Greeting E*	**0.724**	0.170	0.022	-0.183	-0.203
Post-threat interaction, *Greeting E*	**0.687**	0.103	0.288	0.054	-0.041
Obedience, *Latency to obey*	**-0.604**	0.191	0.188	-0.051	-0.002
Ball play, *Playfulness*	**0.507**	-0.163	-0.155	0.201	0.014
T-shirt test, *Moving independently*	0.008	**0.734**	0.099	0.067	0.051
Picture viewing, *Moving independently*	0.031	**0.685**	0.143	-0.212	-0.035
Separation, *Moving*	-0.063	**0.567**	0.095	-0.159	-0.035
Greeting after separation, *Greeting O*	0.066	**-0.554**	0.224	-0.349	0.091
Exploration, *Moving*	0.090	**0.484**	0.061	0.056	0.037
Obedience, *Distractibility*	0.106	0.086	**0.726**	0.201	0.063
Ball play, *Gaze alternation*	0.028	0.057	**0.665**	0.085	-0.021
Problem I (cage), *Orientation to cage*	-0.136	0.141	**0.611**	-0.117	-0.117
Novel object, *Orientation to toy*	-0.020	0.065	**0.535**	**-0.408**	-0.041
Problem I (cage), *Success*	0.009	0.030	0.151	**0.814**	0.085
Problem II (bin), *Success*	0.019	-0.092	-0.058	**0.762**	-0.205
Problem I (cage), *Vocalisation*	0.057	-0.096	0.033	0.115	**-0.812**
Frustration test, *Frustration*	0.124	0.116	0.078	-0.014	**-0.806**
Eigenvalue	2.632	2.275	1.739	1.510	1.306
Explained variance (%)	14.621	12.636	9.663	8.386	7.256

Supplementary Table S3. Descriptive statistics of the seven age groups in Sociability-obedience and Frustration tolerance traits. The mean difference (M2-M1= mean of the younger group subtracted from the mean of the older group), and standardised mean difference (Hedges' g) is calculated from each age group to the next, as well as from late puppyhood to geriatric age (life-long change). In the senior, geriatric, and the life-long change columns, the numbers in brackets represent the values after excluding the four outliers.

	late puppyhood (0.5-1 yr.)	adolescent (>1-2 yr.)	early adult (>2-3 yr.)	middle age (>3-6 yr.)	late adult (>6-8 yr.)	senior (>8-10 yr.)	geriatric (>10 yr.)	life-long change
Sociability-obedience								
N	25	78	26	26	21	19 *(19)*	20 *(17)*	-
mean (M)	0.225	-0.019	-0.252	0.155	0.068	0.111 *(0.077)*	-0.256 *(-0.049)*	-
SD	0.874	1.073	1.221	0.732	0.824	0.799 *(0.808)*	1.170 *(0.924)*	-
M2-M1		-0.244	-0.233	0.407	-0.087	0.043 *(0.009)*	-0.367 *(-0.126)*	-0.481 *(-0.273)*
Hedges' g		-0.237	-0.209	0.404	-0.112	0.053 *(0.011)*	-0.365 *(-0.145)*	-0.474 *(-0.306)*
Frustration tolerance								
N	21	66	23	23	19	18 *(17)*	18 *(15)*	-
mean	-0.353	-0.073	-0.057	0.174	-0.067	0.281 *(0.345)*	0.320 *(0.298)*	-
SD	1.229	1.000	0.698	0.959	0.994	0.884 *(0.868)*	1.154 *(1.267)*	-
M2-M1		0.280	0.016	0.231	-0.241	0.348 *(0.412)*	0.038 *(-0.046)*	0.672 *(0.651)*
Hedges' g		0.265	0.017	0.276	-0.247	0.370 *(0.440)*	0.037 *(-0.043)*	0.563 *(0.523)*
